# Oxidative Stress and Antioxidative Therapy in Pulmonary Arterial Hypertension

**DOI:** 10.3390/molecules27123724

**Published:** 2022-06-09

**Authors:** Dan Xu, Ya-Hui Hu, Xue Gou, Feng-Yang Li, Xi-Yu-Chen Yang, Yun-Man Li, Feng Chen

**Affiliations:** 1 State Key Laboratory of Natural Medicines, Department of Physiology, China Pharmaceutical University, Nanjing 210009, China; 3119090233@stu.cpu.edu.cn (D.X.); 3219091300@stu.cpu.edu.cn (X.G.); yangxyc@stu.cpu.edu.cn (X.-Y.-C.Y.); liyunman@cpu.edu.cn (Y.-M.L.); 2 Department of Pharmacy, Children’s Hospital of Nanjing Medical University, Nanjing 210008, China; 3 Key Laboratory of Chemical Biology (Ministry of Education), Department of Pharmacology, School of Pharmaceutical Sciences, Shandong University, Jinan 250000, China; 202120939@mail.sdu.edu.cn

**Keywords:** pulmonary arterial hypertension, endothelial dysfunction, oxidative stress, therapeutic strategy

## Abstract

Pulmonary arterial hypertension (PAH) is clinically characterized by a progressive increase in pulmonary artery pressure, followed by right ventricular hypertrophy and subsequently right heart failure. The underlying mechanism of PAH includes endothelial dysfunction and intimal smooth muscle proliferation. Numerous studies have shown that oxidative stress is critical in the pathophysiology of PAH and involves changes in reactive oxygen species (ROS), reactive nitrogen (RNS), and nitric oxide (NO) signaling pathways. Disrupted ROS and NO signaling pathways cause the proliferation of pulmonary arterial endothelial cells (PAECs) and pulmonary vascular smooth muscle cells (PASMCs), resulting in DNA damage, metabolic abnormalities, and vascular remodeling. Antioxidant treatment has become a main area of research for the treatment of PAH. This review mainly introduces oxidative stress in the pathogenesis of PAH and antioxidative therapies and explains why targeting oxidative stress is a valid strategy for PAH treatment.

## 1. Introduction

**Figure 1:**
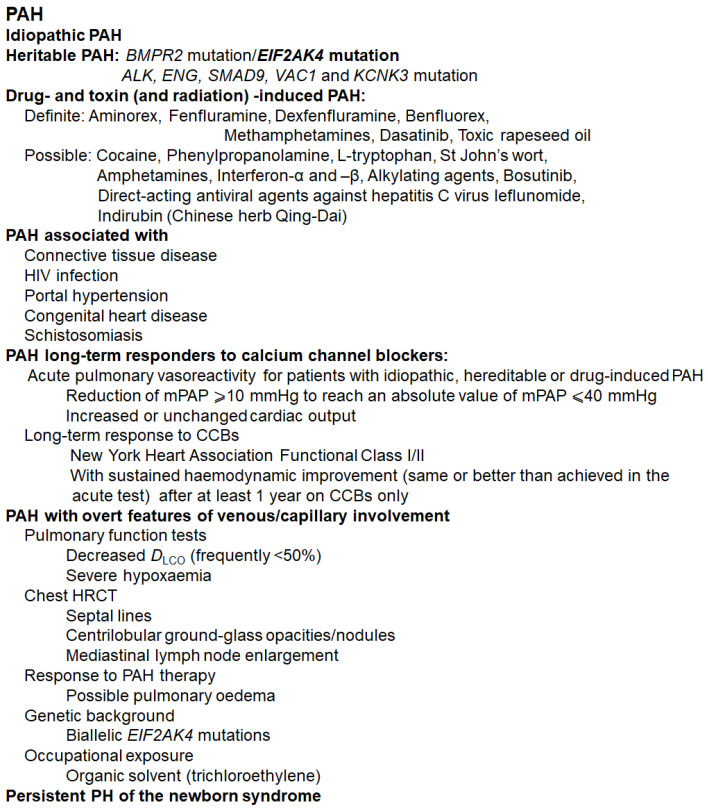
Current classification of PAH. Abbreviations: CCB, calcium channel blocker; mPAP, mean pulmonary arterial pressure; PVOD, pulmonary veno-occlusive disease; PCH, pulmonary capillary hemangiomatosis; DLCO, diffusing capacity of the lung for carbon monoxide; HRCT, high-resolution computed tomography; BMPR, bone morphogenic protein receptor type II.; ALK1, activin receptor-like kinase 1; ENG, endoglin; CAV1, caveolin-1; KCNK3, potassium channel, two-pore domain subfamily K member 3; and EIF2AK4, eukaryotic translation initiation factor 2α kinase 4 [2,4,5,6,8,10].

Pulmonary hypertension (PH) is defined as a resting mean pulmonary artery pressure of 25 mm Hg or above that affects the arteries in the lungs of patients and the right side of the heart. Epidemiological studies have shown that the 5-year overall survival rate of patients with PAH is only 59%. Data demonstrate that if left untreated, numerous patients with PAH will die within two to three years after diagnosis. Accurate diagnosis and classification are key to the overall survival rate [1]. The World Health Organization (WHO) categorizes PH into five groups. Pulmonary arterial hypertension (PAH) is Class I among these groups, with pulmonary arterial (PA) wedge pressure (PAWP) ≤15 mmHg and pulmonary vascular resistance (PVR) >240 dyn × s × cm^−5^ [2]. The main characteristic of PAH is obstructive remodeling of the pulmonary vascular bed, which causes persistent elevation of the mean PA pressure at rest, pulmonary vascular resistance, and, ultimately, right heart failure [3]. Pulmonary vascular remodeling in PAH is not only an accumulation of different vascular cells in the PA wall (e.g., pulmonary artery smooth muscle cells (PASMCs), endothelial cells (ECs), fibroblasts, myofibroblasts, and pericytes) but also a loss of precapillary arteries and perivascular infiltration of inflammatory cells, including B- and T-lymphocytes, mast cells, dendritic cells, and macrophages. PAH is divided into seven subgroups [4,5,6,7]: idiopathic PAH; heritable PAH; drug- and toxin- (and radiation-) induced PAH [8]; PAH associated with various conditions, including connective tissue diseases, HIV infection, portal hypertension, and congenital heart disease; PAH in long-term responders to calcium channel blockers; PAH with venous/capillary involvement; and persistent PH of newborns [9] (see Figure 1).

**Figure 2:**
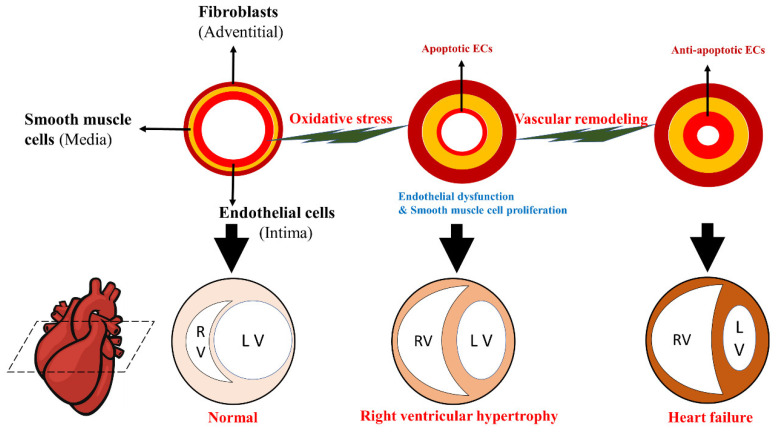
Schematic progression of pulmonary arterial hypertension. Progressive vascular remodeling associated with PA cell proliferation and apoptosis resistance occurs in the distal pulmonary artery after endothelial dysfunction. This structural change gradually results in pulmonary lumen occlusion and increases pulmonary vascular resistance (PVR) and PA pressure. Due to pressure overload, the right ventricle (RV) initially compensates through hypertrophy and increased contractility to maintain cardiac output, gradually leading to heart failure and eventual death.

Pulmonary arterial endothelial cell (PAEC) dysfunction is considered the leading cause of PAH events [11]. Dysfunction of PAECs plays a vital role in the pathogenesis of PAH through impaired vasoconstriction involved in NO signaling, prostacyclin and endothelin, endothelial cell proliferation imbalance, abnormal endothelial-mesenchymal transition, altered production of endothelial vasoactive mediators, altered ion homeostasis, ion channel dysfunction, and epigenetic disorders [12]. Apoptosis occurs in PAECs due to genetic susceptibility and exposure to various kinds of damage (e.g., extreme oxidation emergency, shear stress, and inflammation) [13]. The initial apoptosis of ECs may induce the release of mediators for the proliferation of vascular SMCs, and apoptotic ECs may lose regulatory control of SMCs, thus leading to the proliferation of SMCs [14]. Accurate control of the balance between PASMC proliferation and apoptosis is of great significance for maintaining the structural and functional integrity of pulmonary vessels. In severe vaso-proliferative PH, this balance is disrupted, accompanied by increased PASMC proliferation and a decreased apoptosis rate, leading to vascular wall thickening and remodeling, namely, PASMC hyperplasia [15]. Resistance to apoptosis and an increase in the proliferation rate of PASMCs seem to be necessary for the formation of new intima [11] and are core processes of vascular remodeling. Abnormal levels of growth factors or cytokines produced by ECs and SMCs may also play autocrine or paracrine roles in promoting the progression of PA remodeling, finally leading to PAH [16] with progressive narrowing or complete occlusion of the vascular lumen (see Figure 2).

## 2. Oxidative Stress, DNA Damage, and Oxidative Metabolism in PAH

Oxidative stress reflects an imbalance between the systemic manifestation of ROS and a biological system’s ability to readily detoxify the reactive intermediates or repair the resulting damage. ROS and reactive nitrogen species (RNS) are free radicals closely related to oxidative stress [17], including singlet oxygen (O_2_), hydroxyl radical (OH), superoxide anion (O_2_), hydrogen peroxide (H_2_O_2_), nitric oxide (NO), and peroxynitrite (ONOO-).

The stable state of ROS concentration is determined by the interaction between their production and ROS defense enzymes [18]. Low levels of mitochondrial ROS are thought to play critical roles in signal transduction mechanisms. ROS produced by respiratory chain complex I mainly cause oxidative damage, while ROS produced by complex III are mainly involved in cell signal transduction [19]. There are multiple pathways for intracellular ROS production [20], including the superoxide anions produced by the mitochondrial respiratory chain [21], the NO synthase (NOS) uncoupling process, and nicotinamide adenine dinucleotide phosphate (NADPH) oxidases (Nox), which accept electrons from oxygen. Nox comprises seven subtypes, i.e., Nox 1–7, and Nox4 was recently found to be a major source of oxidative stress [22,23].

DNA damage is increased in human PAH lungs, remodeled arteries, PASMCs, and PAECs [24]. ROS and DNA damage are both biomarkers of PAH susceptibility in multiple PAH subgroups. DNA damage in PASMCs is associated with excessive expression of poly (ADP-ribose) polymerase 1 (PARP1), provirus integration site (PIM1), eyes absent homolog-3 (EYA-3), checkpoint kinase 1 (CHK-1) and surviving genes [25]. In PAECs, BMPR2 downregulation can reduce breast and ovarian cancer susceptibility protein 1 (BRCA1) expression and increase susceptibility to DNA damage. The reduced expression of PPARγ and BMPR2 in PAH-PAECs led to downregulation of the downstream homologous recombination DNA repair genes ataxia telangiectasia mutated (ATM) and RAD51, respectively [26]. In addition, mutations in topoisomerase DNA II-binding protein 1 (TOPBP1) are also associated with PAH susceptibility, maintenance of genomic integrity, prevention of DNA damage during replication, and promotion of PAEC amplification and apoptosis resistance [25].

Elevated ROS levels also lead to increased amounts of mitochondrial DNA (mtDNA) damage [27]. Mitochondria are more sensitive to DNA damage than nuclear DNA because mtDNA lacks the protection of histones. In addition, mtDNA damage repair in PAECs was slower than that in pulmonary venous ECs and microvascular ECs, suggesting that mtDNA damage may be related to PAH [24].

In PAH-PAECs, *BMPR2* downregulation is related to decreased expression of *Tfam* (mtDNA maintenance gene, i.e., transcription factor A, mitochondrial) but increased damage to mtDNA and increased glycolysis, which triggers the dysfunction of PAH-PAECs in the endothelium. In PAH-PASMCs, mitochondrial HSP90 accumulation increases with upregulated *POLG1* (mitochondrial DNA polymerase γ) and *OG1* (8-oxoguanine glycosylase). This is believed to be a regulatory mechanism to maintain mtDNA and metabolic reprogramming under stress conditions and to promote the survival of PAH-PASMCs [28].

PAH also leads to abnormalities in oxidative metabolism [29]. Cells affected by PAH acquire a Warburg phenotype characterized by mitochondrial hyperpolarization, a decrease in pyruvate dehydrogenase complex activity, and a reduction in mitochondrial ROS levels. This seems to be contrary to the elevated oxidative stress in PAH, which has not been reported in detail thus far. Many studies have shown that DNA methylation, which epigenetically silences SOD2, especially in PAH patients, leads to a reduction in H2O2 production and activation of HIF-1α [30], thereby destroying mitochondrial metabolism and dynamics, increasing aerobic glycolysis, and resulting in accelerated PASMC proliferation and inhibited apoptosis [26].

## 3. Oxidative Stress Signal Transduction in Pulmonary Hypertension Vascular Remodeling

The NOS family catalyzes the production of NO from L-arginine. The arginine-NOS-NO pathway is important in the regulation and remodeling of PAH vascular tension. Recent studies have shown that the carbonic anhydrase 1-kininogen and selenium protein W/14-3-3 signaling pathway attenuates the inhibitory effect on eNOS, promotes NO production, and regulates oxidative stress in Monocrotaline (MCT)-induced rat models [31]. During the first week in the MCT model, nitrosative stress leads to adaptation of NOS activity to later increase NO production after two weeks. In the third week, oxidative stress became very obvious [32]. The accumulation of superoxide anions causes NO content decline and the uncoupling of eNOS [33], where electrons are transferred from the NOS reductase domain of dysfunctional enzymes to an oxygenase domain and to molecular oxygen instead of L-arginine, eventually forming superoxide but not NO [34].

## 4. Oxidative Stress-Induced Dysfunction of Pulmonary Artery ECs

Pulmonary vascular endothelial dysfunction is associated with reduced bioavailability of NO and increased degradation of NO due to oxidative stress [35,36]. Human pulmonary ECs mainly express superoxides producing Nox1 and Nox2 and hydrogen peroxide/superoxide producing Nox4 [37]. Nox-mediated oxidative stress produces superoxide anions, and pulmonary endothelial dysfunction is closely related to PH [38]. Oxidative stress can aggravate endothelial dysfunction, characterized by an increase in the synthesis and release of endothelium-derived contraction factors (such as endothelin 1 and ET-1) and a decrease in diastolic factors (such as NO) [32].

Under hypoxic and oxidative stress, cells can secrete cyclophilin A (CyPA, especially its acetylated form) and participate in the induction and regulation of EC autophagy in PH, where a positive feedback relationship relies on autophagy and Nox activity [39,40]. Recent studies have revealed that extracellular CyPA promotes interstitial transformation, migration, and proliferation in ECs, leading to mitochondrial dysfunction and oxidative stress [41]. The PAH rat model exposed to 10% hypoxia for 3 weeks showed apoptosis of endothelial progenitor cells (EPCs), an increase in Nox and vascular peroxidase (VPO1) expression, and increased H_2_O_2_ and hypochlorite levels. These effects could be attenuated by Nox2 or Nox4 siRNA, demonstrating that ROS derived from the Nox/VPO1 pathway can promote oxidative damage in EPCs [42]. After hypoxia exposure to pulmonary ECs, Nox1-derived ROS generation upregulated the expression of the endogenous BMP antagonist Gremlin1 by activating the proangiogenic factor Sonic hedgehog, leading to excessive proliferation of pulmonary ECs and PAH [43]. Other factors, such as Kruppel-like factor 4 (KLF4), also play a role in the pathogenesis of PAH vascular dysfunction [44].

## 5. Oxidative Stress Signaling-Associated Proliferation of PASMCs

Evidence has shown that ROS may regulate cellular signal transduction, especially when Nox is the main source of ROS. The inducing role of Nox in the proliferation of PASMCs has been demonstrated in many PAH models, and it may be related to the downregulation of the Keap-1/Nrf2 pathway. Nox4-induced ROS triggers PAH; however, new research also claimed that Nox4 may promote homeostasis pathways in PASMCs, allowing cell survival and adaptation to ERS [45]. Selenoprotein P in the lung can decrease the production of glutathione, increase the production of GSSG, upregulate the production of Nox-induced ROS, and stabilize HIF-1α, thus leading to mitochondrial dysfunction, proliferation of PAH-PASMCs, and apoptosis [46].

## 6. Oxidative Stress and Its Signal Transduction in Right Ventricular Hypertrophy

Right ventricular hypertrophy due to PAH can lead to right ventricular failure (RVF) and eventually to death [47]. This process is largely associated with oxidative stress and partly due to the inability to upregulate MnSOD in the right ventricle [48]. In chronic thromboembolic pulmonary hypertension, Nox1, Nox2, and Nox4 were increased in the right coronary artery, accompanied by oxidative stress and endothelial dysfunction, but eNOS expression remained unchanged [49]. Dysfunctional mitochondria also release additional ROS, aggravate oxidative stress status, and lead to MCT- and hypoxia-induced RVF in PAH rats [50]. On the other hand, lack of NOS_2_ and NO induction prevented superoxide scavenging, decreased reactive oxidant formation (ONOO^2−^), and improved adaptation of the right ventricle to PAH [51].

PAH is associated with decreased bioavailability of NO and increased asymmetric dimethyl arginine (ADMA), an endogenous NOS inhibitor. Dimethylarginine dimethylaminohydrolase 1 (DDAH1) knockout rats showed significantly increased plasma and pulmonary ADMA content and decreased eNOS protein content and NO release, aggravating oxidative stress, pulmonary vascular remodeling, and fibrosis in MCT-induced PAH rats. This study indicated that DDAH1 degrades ADMA and protects the right ventricle from hypertrophy in PAH rats [52]. Protein kinase G (PKG)-Iα is oxidized to a dithionin-activated state by Nox-4, SOD3, cystathionine γ-lyase, etc., and its expression is increased in the lungs of PAH patients and in hypoxic PAH mice. PKG-Iα is involved in endogenous and adaptive redox mechanisms by promoting vasodilation, thus limiting the remodeling caused by PAH and related adverse pulmonary artery and right heart remodeling, making it another important target for PAH treatment [53]. 

## 7. Oxidative Stress Is Involved in the Formation of Pulmonary Hypertension through Growth Factors and Their Signal Transduction Systems

ROS promotes the expression and/or activation of growth factors, including p38 mitogen-activated protein kinase (MAPK), nonreceptor tyrosine kinase c-SRC, TGF-β1, VEGF, fibroblast growth factor 2 (FGF2), platelet-derived growth factor (PDGF), and peroxisome proliferators-activated receptor-γ-coactivator-1 (PGC-1α) [54]. In addition, growth factors also stimulate the production of ROS. Oxidative stress promotes the endothelial-to-mesenchymal transition of PAHs by activating the TGF-β pathway [41]. Nox4 mediates TGF-β1-dependent pulmonary vascular remodeling and PDGF and HIF-1α activities. The multidirectional interactions between Nox-derived ROS, HIF-1α, and TGF-β1 may be the key to the pathogenesis of PAH [55]. Growth differentiation factor-15 (GDF-15) is a multifactor cytokine and a member of the TGF superfamily. GDF-15 gene expression in cardiomyocytes, vascular SMCs, and ECs leads to significantly associated oxidative stress, inflammation, and tissue damage. GDF-15 is highly expressed in the vascular chambers of patients with PAH and hypoxia and may be a new indicator of PH [56]. In vascular SMCs, c-Src is also necessary for ROS production. The binding of AT-II to the AT1 receptor triggers Nox-dependent O_2_ production, which leads to phosphorylation and activation of EGFR. In PASMCs, H_2_O_2_-mediated oxidation stimulates intracellular intrinsic protein tyrosine kinase activity and the formation of the covalently modified EGFR dimer. Similar to EGFR, PDGFR promotes the proliferation and migration of PASMCs during PAH development by activating Nox4-induced abnormal oxidative stress, which can be effectively eliminated by CD248 knockout or antibody therapy [57]. In addition, PDGF promotes apoptosis resistance of pulmonary arterial ECs and the development of endothelial plexus lesions [58]. VEGF binds to the tyrosine kinase receptor (VEGFR-1/VEGFR-2) located in the vascular endothelium with high affinity. VEGF overexpression in neonatal mice induces NOS (iNOS), eNOS-dependent pulmonary edema, and oxidative stress. In VEGF-transgenic mice, NOS inhibition has been shown to reduce oxidative stress [59].

## 8. General Advancements in the Antioxidant Treatment of PAH

### 8.1. Current PAH Treatments

There is no cure for PAH, but treatments are available to control symptoms and improve quality of life. Current medicines for PAH treatment include endothelin receptor antagonists (e.g., bosentan and ambrisentan), prostacyclin analogs (e.g., epoprostenol and misoprostol), agonists of the prostacyclin receptor (e.g., selexipag), phosphodiesterase (PDE-5) inhibitors (e.g., sildenafil and vardenafil), angiotensin II, and the sGC stimulator (e.g., riociguat) [35,60].

### 8.2. Latest Strategies for Oxidative Stress in PAH

#### 8.2.1. Preclinical Strategies: Antioxidants

Table 1 shows the very recent advances in medicines targeting oxidative stress. Alginate oligoside (AOS) acts as an antioxidant and anti-inflammatory agent in PAH by inhibiting MCT-induced pulmonary vascular remodeling. AOS blocks the TGF-β1/p-Smad2 signaling pathway, downregulates the expression of MDA, Nox, and proinflammatory cytokines, reduces macrophage infiltration, and upregulates the expression of anti-inflammatory cytokines [61]. Sulforaphane upregulates the expression of Nrf2 and its downstream gene NQO1 and reduces SuHx-induced pulmonary vascular remodeling, inflammation, and fibrosis [62]. As a natural antioxidant, ellagic acid has a protective effect on PAH and lung and heart injury in SD rats caused by porcine pancreatic elastase, which reduces antioxidant levels [63]. Oral melatonin administered to maternal sheep in the late gestation period improved the pulmonary vascular function of plateau PAH lambs, enhanced antioxidant capacity, and reduced the production of ROS and nitrotyrosine, a marker of oxidative stress in small pulmonary vessels [64,65]. 18β-Glycyrrhetinic acid downregulated MDA levels, improved SOD, CAT, T-AOC, and GSH-PX functions, and inhibited PAH-induced oxidative stress in rats [66]. Oral administration of the ASK1 inhibitor GS-444217 (or selonsertib) reduced pulmonary arterial pressure and reduced right ventricular hypertrophy in the PAH model in a dose-dependent manner [67,68]. PIM1 phosphorylates KU70 and enhances nonhomologous end-joining DNA repair in PAH, and its pharmacological inhibitors SGI-1776 and TP-3654 attenuate PAH in MCT and fawn-hooded rat models [69]. Celastramycin increases the protein level of Nrf2, enhances mitochondrial energy metabolism, and restores mitochondrial network formation in PASMCs [70]. Celastrol inhibits the expression of CyPA and Bsg in the heart and lungs, thereby improving heart failure and postcapillary PAH in mice [69]. In addition, it was shown that exercise training alleviated oxidative stress in the gastrocnemius of rats with MCT-induced PAH [71].

Combination therapy has become a bellwether for PAH treatment. Novel hybridization of isosorbide 5 mononitrate and bardoxolone methyl inhibited excessive proliferation of perivascular cells, reduced macrophage infiltration, and oxidative stress, and exerted inhibitory effects on double vasodilation and vascular remodeling in rat PAH models [77]. Combined administration of chloroacetic acid (DCA) and atorvastatin (ATO) upregulated oxidative stress, reduced PASMC activity, and reduced mitochondrial membrane potential in MCT rat models [78,92]. 

#### 8.2.2. Preclinical Strategies: Herbal Treatments

Natural herbs and plants also offer many new strategies that target oxidative stress in PAHs. Crocin exerted a protective effect on PAH induced by MCT in rats by regulating the oxidation resistance 1 (OXR1) signaling pathway [74]. Ocimum sanctum (Linn) reduced the expression of thiobarbiturate reactants and Nox1, promoted the release of catalase, and had a beneficial effect on MCT-induced PH in rats [91]. Blueberry extracts reduced the activity of Nox and XO expression, improved the activity of SOD, restored sulfhydryl content and the expression of Nrf2, and increased the expression ratio of ETA/ETB in the lungs of rat PAH models [90]. Honokiol alleviates CyPA-regulated autophagy and PAH in pulmonary arterial ECs [39].

#### 8.2.3. Clinical Trials

Emulsion of N-3 long-chain polyunsaturated fatty acids (LC-PUFAs) plays an important role in fetal and infant growth and development. A recent clinical study (NCT04031508) on neonatal PPHN was conducted to explore potential changes in markers of inflammation and oxidative stress. Olaparib is currently being studied in an open-label, single-arm study with a primary endpoint of changes in PVR at 16 weeks (NCT03251872) [93] for its role in DNA damage and PARP inhibition. A current crossover trial is investigating whether dehydroepiandrosterone (DHEA), which has direct effects on NO and ET-1 synthesis and signaling, direct antihy-pertrophic effects on cardiomyocytes, and mitigates oxidative stress [94] can be used to measure right ventricular longitudinal tension by cardiac magnetic resonance imaging at 18 weeks compared with placebo and to evaluate its side effects and safety in patients with PH (NCT03648385). Olaparib is an oral PARP inhibitor and has been approved for the treatment of ovarian cancer in Canada, Europe, and the United States. Currently, the safety of olaparib in PAH patients is being investigated, and the sample size in future phase 2 trials is being refined to assess its efficacy (NCT03782818). There is evidence showing that bromodomain-containing protein 4 (BRD4, an upstream target protein of Keap1/Nrf2 and a key regulator of oxidative stress in a variety of cell types [95]) plays a key role in the pathologic phenotype of PAH. In some animal models, BRD4 inhibition can reverse PAH. Apabetalone is an oral BRD4 inhibitor that is currently in clinical development and has a suitable safety profile (NCT03655704). A proof-of-concept phase II clinical trial was recently completed in which researchers evaluated a first-in-class ASK1 (Apoptosis signal-regulating kinase-1 (ASK1), a member of the mitogen-activated protein (MAP) kinase family that is activated by oxidative stress and promotes inflammation and apoptosis [96]) inhibitor (selonsertib) in 151 adult patients with PAH (NCT02234141). Participants were randomized to receive 2, 6, or 18 mg selonsertib tablets once daily for 24 weeks during the treatment phase (Period 1) and may have continued on this dosing during the long-term treatment phase (Period 2). However, the last results provided in 2019 were disappointing due to no obvious efficacy compared with the placebo group.

## 9. Conclusions

**Figure 3:**
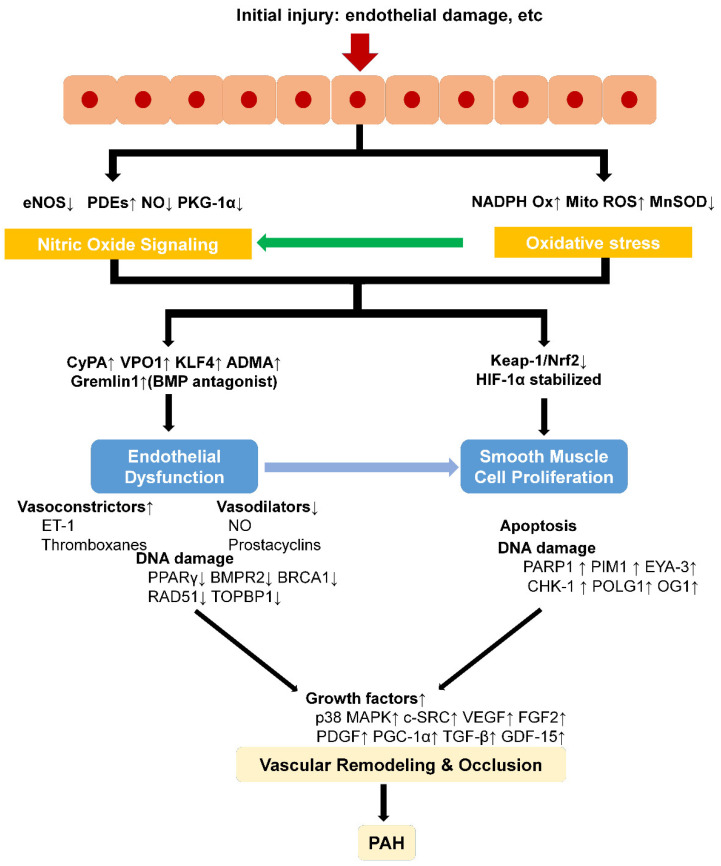
Schematic mechanisms of oxidative stress in PAH. Abbreviations: PDE, phosphodiesterase-5, PKG-Iα, protein kinase G-Iα, CyPA, cyclophilin A, VPO1, vascular peroxidase1, KLF4, Kruppel-like factor 4, ADMA, asymmetric dimethyl arginine, BMP, bone morphogenic protein, BRCA1, breast and ovarian cancer susceptibility protein 1, TOPBP1, topoisomerase DNA II-binding protein 1, PARP1, poly (ADP-ribose) polymerase 1, PIM1, provirus integration site, EYA-3, eyes absent homolog-3, CHK-1, checkpoint kinase 1, POLG1, mitochondrial DNA polymerase γ, OG1, 8-oxoguanine glycosylase, FGF2, fibroblast growth factor 2, PDGF, platelet-derived growth factor, PGC-1α, peroxisome proliferators-activated receptor-γ-coactivator-1, GDF-15, growth differentiation factor-15.

In summary, PAH is associated with a generalized state of enhanced oxidative stress (Figure 3). Oxidative stress is involved in the formation and development of PAH in many ways, including pulmonary vascular remodeling, dysfunction of pulmonary ECs, proliferation of PASMCs, and right ventricular hypertrophy, at which the development of treatments for PAH targeting oxidative stress is mainly aimed. The development of therapy for PAH targeting oxidative stress is still an open and hot field with no perfect preclinical model to fully replicate human PAH injury and no satisfying and precise treatment. Therefore, we need more relevant targets and thorough preclinical and clinical research.

## Figures and Tables

**Table 1:** Preclinical treatments and mechanisms targeting oxidative stress in PAH.

Drugs	Type of Drug	Animals	Model	Biological Indicators	Administration	Therapeutic Effect	References
Alginate Oligosaccharide (AOS)	biodegradable polymer	Sprague-Dawley rats	MCT (i.p.)	p47-phox, p67-phox, and gp91-phox, subunits of NADPH oxidase, MDA	i.p.	Down-regulate the expressions of malondialdehyde and NADPH by inhibiting the TGF-β1 /p-Smad2 signaling pathway to prevent the pulmonary vascular remodeling induced by MCT	[61]
Vardenafil	Phosphodiesterase-5 inhibitor	Sprague-Dawley rats	MCT (i.p.)	8-iso-prostaglandin-F2a, 3-nitrotyrosine, eNOS, NO, MDA, SOD, Nox2, Nox4	i.g.	Suppress proliferation and enhanced apoptosis of pulmonary artery smooth muscle cells, attenuating small pulmonary artery remodeling, and right ventricular hypertrophy	[72]
Pentaerythritol Tetranitrate	——	Wistar rats	MCT (i.v.)	HO-1, ICAM-1	i.g.	PETN therapy improved endothelium-dependent relaxation in pulmonary arteries and reduced oxidative stress	[73]
Sulforaphane	Nrf2 activator	Male mice	SU5416 and 10% hypoxia (SuHx)	Nrf2, NQO1, NLRP3	i.g.	Reduce SuHx-induced pulmonary vascular remodeling, inflammation, and fibrosis	[62]
Crocin	——	Sprague-Dawley rats	MCT (i.p.)	OXR1, P21, Nrf2	i.p.	Crocin co-treatment significantly improved the hemodynamic, oxidative stress biomarkers and histological data of the PAH rats	[74]
Melatonin	——	Newborn sheep	Chronic hypobaric hypoxia (Putre, 3600 m)	SOD2, CAT, GPx1, VDAC, p47-phox, Xantine Oxidase, 8-isoprostanes, 4HNE, and nitrotyrosine	i.g.	Reduced major sources of pro-oxidative ROS at the cellular level, reduced oxidative stress and enhanced antioxidant status at the pulmonary level of neonatal PAH	[64,65]
Resveratrol	Polyphenolic compound	Sprague-Dawley rats	Hypoxia	Nrf2, HIF-1 α	i.g.	Exert antiproliferation, antioxidant, and anti-inflammation effects	[75]
Ellagic Acid	——	Male Sprague-Dawley rats	Porcine pancreatic elastase(intratracheal)	SOD, catalase, and glutathione	i.g.	Reduce oxidative stress and prevent PAH	[63]
18β-Glycyrrhetinic Acid	——	Male Sprague-Dawley rats	MCT (i.p.)	Nox2, Nox4	i.g.	Reduce the changes in oxidative stress biomarkers and inhibit Nox2 and Nox4 expression	[66]
Celastramycin	——	Wild-type mice; SD rats	3 wk of hypoxic exposure (10% O2); SU5416, s.c.	ROS, Nrf2, Nox, GSH/GSSG, SOD2	Osmotic pump; i.p.	Increase protein levels of Nrf2 (nuclear factor erythrocyte-related factor 2) and improve pulmonary hypertension	[70]
Celastrol	Tripterygium wilfordii extractive	*cROCK1*^−/−^ and *cROCK2*^−/−^ mice	TAC	CyPA, Bsg, Nox2, Nox4	i.p.	Inhibit CyPA/Bsg-NF-κB axis and enhance ROS production	[76]
Hybridization of Isosorbide 5 Mononitrate and Bardoxolone Methyl	A NO donor and a semisynthetic derivative of oleanolic acid	Male Sprague-Dawley rats	MCT (i.h.)	NO, Nox4	i.t.	By inactivating Nox4, excessive proliferation of vascular pericytes was inhibited, macrophage infiltration and oxidative stress were reduced, and cardiac hypertrophy and fibrosis were significantly reduced in rats with pulmonary hypertension	[77]
Combination of Dichloroacetate and Atorvastatin	——	Male Sprague-Dawley rats	MCT (i.h.)	CHOP, Bcl2	i.g.	The combined treatment of DCA/ATO significantly reduces the right ventricular systolic blood pressure accompanied by a decrease in right heart hypertrophy and reduces vascular remodeling, thereby inhibiting excessive PASMC proliferation	[78]
Baicalein	Natural flavonoid	Male Sprague-Dawley rats	MCT (i.h.)	MDA, SOD, GSH-Px, Bax, Bcl-2	i.g.	Inhibit oxidative stress and alleviated pulmonary vascular remodeling in MCT-induced PAH	[79]
17-β estradiol	Estrogen	Male Sprague-Dawley rats	MCT (i.h.)	T-AOC, MDA, Nox4	i.p.	Inhibit Nox4-mediated oxidative stress and alleviated MCT-induced right ventricular remodeling of PAH rats	[80]
Copaiba Oil	——	Male Wistar rats	MCT (i.p.)	eNOS	i.g.	Reduce oxidative stress and apoptosis signaling in RV of rats with PAH	[81]
Dimethyl Fumarate	Antioxidative and anti-inflammatory agent	Male C57BL/6 mice	Hypoxic chamber	HO-1, NOX4	i.p.	Mitigate oxidative stress damage and inflammation in lung	[82]
Bucindolol	β-adrenergic blocker	Male Wistar rats	MCT (i.p.)	eNOS, SOD-1	i.p.	Decrease (21%) PVR and increase RV workload, thereby improving the vascular remodeling of the pulmonary artery	[83]
Rosuvastatin	——	Male Ren2 and Sprague-Dawley rats	Transgenic (mRen2) 27 rats	3-NT, NO(x), Nox, and endothelial NO synthase expression	i.p.	Improve cardiovascular outcomes/risk by restoring endothelial and SMC function, inhibiting SMC proliferation, reducing oxidative stress and inflammation in the vascular wall	[84]
Carvacrol	——	Male Wistar rats	Hypoxia	SOD, GSH, MDA, caspase-3	i.p.	Attenuate the pulmonary vascular remodeling and promotes PASMC apoptosis	[85]
Trapidil	——	Male Wistar rats	MCT (i.p.)	NADPH oxidases, glutathiones/total glutathiones	i.p.	Improve hemodynamic, echocardiographic, and redox state parameters of right ventricle	[86]
Tetrandrine	Bisbenzylisoquinoline alkaloid	Male Sprague-Dawley rats	MCT (i.p.)	cGMP, PKG-1, iNOS	i.p.	Alleviate MCT-induced PAH through regulation of NO signaling pathway and antioxidant and antiproliferation effects	[87]
Trimethoxystilbene	Resveratrol analog	Male Sprague-Dawley rats	Hypoxic chamber	Nox2, Nox4, VPO1	i.g.	Attenuate hypoxia-induced pulmonary vascular remodeling and right ventricle hypertrophy accompanied by downregulation of Nox2, Nox4, and VPO1 expression	[88]
Hydrogen	——	Male Sprague-Dawley rats	MCT (i.h.)	STAT3, NFAT	Housed ad libitum to hydrogen-saturated water	Ameliorate MCT-induced PAH in rats by suppressing macrophage accumulation, reducing oxidative stress, and modulating the STAT3/NFAT axis	[89]
Blueberry extract	herb	Male Wistar rats	MCT (i.p.)	NADPH, SOD, GPx, ET_A_/ET_B_	i.g.	Decrease the mean pulmonary artery pressure and total reactive species concentration and lipid oxidation	[90]
Ocimum Sanctum (Linn)	herb	Male Wistar rats	MCT (i.h.)	Thiobarbituric Acid Reactive Substances (TBARS); GSH; Catalase; SOD; Nox1	i.g.	Decrease Nox-1 expression and increase expression of Bcl2/Bax ratio caused by MCT	[91]
Honokiol	herb	Male Sprague-Dawley rats	MCT (i.p.)	CyPA	i.g.	Alleviate autophagy and PAH regulated by CyPA in PAECs	[39]
GS-444217/Selonsertib	ASK1 inhibitor	SD rats	MCT (i.h)/Sugen/hypoxia	phosphorylation of p38 and JNK	i.g.	Reduce pulmonary arterial pressure and RV hypertrophy in PAH models associated with reduced ASK1 phosphorylation, reduced muscularization of the pulmonary arteries, and reduced fibrotic gene expression in the RV	[67]
SGI-1776, TP-3654	Pim1 inhibitor	Male SD rats	MCT (i.h)/Fawn-Hooded Rats (FHR)	Repair of DNA damage	i.g.	Improve significantly pulmonary hemodynamics (right heart catheterization) and vascular remodeling (Elastica van Gieson)	[69]

## References

[1] Galiè N., Humbert M., Vachiery J., Gibbs S., Lang I., Torbicki A., Simonneau G., Peacock A., Vonk Noordegraaf A., Beghetti M. (2016). 2015 ESC/ERS Guidelines for the diagnosis and treatment of pulmonary hypertension: The Joint Task Force for the Diagnosis and Treatment of Pulmonary Hypertension of the European Society of Cardiology (ESC) and the European Respiratory Society (ERS): Endorsed by: Association for European Paediatric and Congenital Cardiology (AEPC), International Society for Heart and Lung Transplantation (ISHLT). Eur. Heart J..

[2] Hoeper M.M., Ghofrani H.A., Grünig E., Klose H., Olschewski H., Rosenkranz S. (2017). Pulmonary Hypertension. Dtsch. Arztebl. Int..

[3] Bourgeois A., Omura J., Habbout K., Bonnet S., Boucherat O. (2018). Pulmonary arterial hypertension: New pathophysiological insights and emerging therapeutic targets. Int. J. Biochem. Cell Biol..

[4] Foshat M., Boroumand N. (2017). The Evolving Classification of Pulmonary Hypertension. Arch. Pathol. Lab. Med..

[5] Sahay S. (2019). Evaluation and classification of pulmonary arterial hypertension. J. Thorac. Dis..

[6] Coons J.C., Pogue K., Kolodziej A.R., Hirsch G.A., George M.P. (2019). Pulmonary Arterial Hypertension: A Pharmacotherapeutic Update. Curr. Cardiol. Rep..

[7] Simonneau G., Montani D., Celermajer D.S., Denton C.P., Gatzoulis M.A., Krowka M., Williams P.G., Souza R. (2019). Haemodynamic definitions and updated clinical classification of pulmonary hypertension. Eur. Respir. J..

[8] Vonk Noordegraaf A., Groeneveldt J.A., Bogaard H.J. (2016). Pulmonary hypertension. Eur. Respir. Rev..

[9] Mandras S.A., Mehta H.S., Vaidya A. (2020). Pulmonary Hypertension: A Brief Guide for Clinicians. Mayo Clin. Proc..

[10] Guo Y., Ahn M.J., Chan A., Wang C.H., Kang J.H., Kim S.B., Bello M., Arora R.S., Zhang Q., He X. (2019). Afatinib versus methotrexate as second-line treatment in Asian patients with recurrent or metastatic squamous cell carcinoma of the head and neck progressing on or after platinum-based therapy (LUX-Head & Neck 3): An open-label, randomised phase III trial. Ann. Oncol..

[11] Dai Z., Zhu M.M., Peng Y., Jin H., Machireddy N., Qian Z., Zhang X., Zhao Y. (2018). Endothelial and Smooth Muscle Cell Interaction via FoxM1 Signaling Mediates Vascular Remodeling and Pulmonary Hypertension. Am. J. Respir. Crit. Care Med..

[12] Ranchoux B., Harvey L.D., Ayon R.J., Babicheva A., Bonnet S., Chan S.Y., Yuan J.X., Perez V.J. (2018). Endothelial dysfunction in pulmonary arterial hypertension: An evolving landscape (2017 Grover Conference Series). Pulm. Circ..

[13] Le Ribeuz H., Dumont F., Ruellou G., Lambert M., Balliau T., Quatredeniers M., Girerd B., Cohen-Kaminsky S., Mercier O., Yen-Nicolay S. (2020). Proteomic Analysis of KCNK3 Loss of Expression Identified Dysregulated Pathways in Pulmonary Vascular Cells. Int. J. Mol. Sci..

[14] Sakao S., Tatsumi K., Voelkel N.F. (2009). Endothelial cells and pulmonary arterial hypertension: Apoptosis, proliferation, interaction and transdifferentiation. Respir. Res..

[15] Humbert M., Montani D., Perros F., Dorfmüller P., Adnot S., Eddahibi S. (2008). Endothelial cell dysfunction and cross talk between endothelium and smooth muscle cells in pulmonary arterial hypertension. Vasc. Pharm..

[16] Deng L., Blanco F.J., Stevens H., Lu R., Caudrillier A., McBride M., McClure J.D., Grant J., Thomas M., Frid M. (2015). MicroRNA-143 Activation Regulates Smooth Muscle and Endothelial Cell Crosstalk in Pulmonary Arterial Hypertension. Circ. Res..

[17] Zhang C.F., Zhao F.Y., Xu S.L., Liu J., Xing X.Q., Yang J. (2019). Autophagy in pulmonary hypertension: Emerging roles and therapeutic implications. J. Cell. Physiol..

[18] Schlüter K.-D., Kutsche H.S., Hirschhäuser C., Schreckenberg R., Schulz R. (2018). Review on Chamber-Specific Differences in Right and Left Heart Reactive Oxygen Species Handling. Front. Physiol..

[19] Bottje W.G. (2019). Oxidative metabolism and efficiency: The delicate balancing act of mitochondria. Poult. Sci..

[20] Marushchak M., Maksiv K., Krynytska I., Dutchak O., Behosh N. (2019). The Severity of Oxidative Stress in Comorbid Chronic Obstructive Pulmonary Disease (COPD) and Hypertension: Does it Depend On ACE and AGT Gene Polymorphisms?. J. Med. Life.

[21] Perez M., Robbins M.E., Revhaug C., Saugstad O.D. (2019). Oxygen radical disease in the newborn, revisited: Oxidative stress and disease in the newborn period. Free Radic. Biol. Med..

[22] Wu J., Pan W., Wang C., Dong H., Xing L., Hou J., Fang S., Li H., Yang F., Yu B. (2019). H_2_S attenuates endoplasmic reticulum stress in hypoxia-induced pulmonary artery hypertension. Biosci. Rep..

[23] Scioli M.G., Storti G., D’Amico F., Rodriguez Guzman R., Centofanti F., Doldo E., Cespedes Miranda E.M., Orlandi A. (2020). Oxidative Stress and New Pathogenetic Mechanisms in Endothelial Dysfunction: Potential Diagnostic Biomarkers and Therapeutic Targets. J. Clin. Med..

[24] Ranchoux B., Meloche J., Paulin R., Boucherat O., Provencher S., Bonnet S. (2016). DNA Damage and Pulmonary Hypertension. Int. J. Mol. Sci..

[25] Sharma S., Aldred M.A. (2020). DNA Damage and Repair in Pulmonary Arterial Hypertension. Genes.

[26] Leopold J.A., Maron B.A. (2016). Molecular Mechanisms of Pulmonary Vascular Remodeling in Pulmonary Arterial Hypertension. Int. J. Mol. Sci..

[27] Wauchope O.R., Mitchener M.M., Beavers W.N., Galligan J.J., Camarillo J.M., Sanders W.D., Kingsley P.J., Shim H.N., Blackwell T., Luong T. (2018). Oxidative stress increases M1dG, a major peroxidation-derived DNA adduct, in mitochondrial DNA. Nucleic Acids Res..

[28] Boucherat O., Peterlini T., Bourgeois A., Nadeau V., Breuils-Bonnet S., Boilet-Molez S., Potus F., Meloche J., Chabot S., Lambert C. (2018). Mitochondrial HSP90 Accumulation Promotes Vascular Remodeling in Pulmonary Arterial Hypertension. Am. J. Respir. Crit. Care Med..

[29] Wu D., Dasgupta A., Read A.D., Bentley R.E.T., Motamed M., Chen K.H., Al-Qazazi R., Mewburn J.D., Dunham-Snary K.J., Alizadeh E. (2021). Oxygen sensing, mitochondrial biology and experimental therapeutics for pulmonary hypertension and cancer. Free Radic. Biol. Med..

[30] Archer S.L., Marsboom G., Kim G.H., Zhang H.J., Toth P.T., Svensson E.C., Dyck J.R., Gomberg-Maitland M., Thebaud B., Husain A.N. (2010). Epigenetic attenuation of mitochondrial superoxide dismutase 2 in pulmonary arterial hypertension: A basis for excessive cell proliferation and a new therapeutic target. Circulation.

[31] Yu W.-C., Chen H.-Y., Yang H.-L., Xia P., Zou C.-W., Sun T.-W., Wang L.-X. (2019). rBMSC/Cav-1F92A Mediates Oxidative Stress in PAH Rat by Regulating SelW/14-3-3η and CA1/Kininogen Signal Transduction. Stem Cells Int..

[32] Zimmer A., Teixeira R.B., Constantin R.L., Campos-Carraro C., Aparicio Cordero E.A., Ortiz V.D., Donatti L., Gonzalez E., Bahr A.C., Visioli F. (2021). The progression of pulmonary arterial hypertension induced by monocrotaline is characterized by lung nitrosative and oxidative stress, and impaired pulmonary artery reactivity. Eur. J. Pharmacol..

[33] Rochette L., Lorin J., Zeller M., Guilland J.C., Lorgis L., Cottin Y., Vergely C. (2013). Nitric oxide synthase inhibition and oxidative stress in cardiovascular diseases: Possible therapeutic targets?. Pharmacol. Ther..

[34] Tabima D.M., Frizzell S., Gladwin M.T. (2012). Reactive oxygen and nitrogen species in pulmonary hypertension. Free Radic. Biol. Med..

[35] Zolty R. (2020). Pulmonary arterial hypertension specific therapy: The old and the new. Pharmacol. Ther..

[36] Crosswhite P., Sun Z. (2010). Nitric oxide, oxidative stress and inflammation in pulmonary arterial hypertension. J. Hypertens..

[37] Agarwal S., Sharma H., Chen L., Dhillon N.K. (2020). NADPH oxidase-mediated endothelial injury in HIV- and opioid-induced pulmonary arterial hypertension. Am. J. Physiol. Lung Cell. Mol. Physiol..

[38] Novelli E.M., Little-Ihrig L., Knupp H.E., Rogers N.M., Yao M., Baust J.J., Meijles D., St Croix C.M., Ross M.A., Pagano P.J. (2019). Vascular TSP1-CD47 signaling promotes sickle cell-associated arterial vasculopathy and pulmonary hypertension in mice. Am. J. Physiol. Lung Cell. Mol. Physiol..

[39] Wang X., Xiao D., Ma C., Zhang L., Duan Q., Zheng X., Mao M., Zhu D., Li Q. (2019). The effect of honokiol on pulmonary artery endothelium cell autophagy mediated by cyclophilin A in hypoxic pulmonary arterial hypertension. J. Pharmacol. Sci..

[40] Ornatowski W., Lu Q., Yegambaram M., Garcia A.E., Zemskov E.A., Maltepe E., Fineman J.R., Wang T., Black S.M. (2020). Complex interplay between autophagy and oxidative stress in the development of pulmonary disease. Redox Biol..

[41] Xue C., Senchanthisai S., Sowden M., Pang J., White J., Berk B.C. (2020). Endothelial-to-Mesenchymal Transition and Inflammation Play Key Roles in Cyclophilin A–Induced Pulmonary Arterial Hypertension. Hypertension.

[42] Wang E.L., Jia M.-M., Luo F.-M., Li T., Peng J.-J., Luo X.-J., Song F.-L., Yang J.-F., Peng J., Liu B. (2019). Coordination between NADPH oxidase and vascular peroxidase 1 promotes dysfunctions of endothelial progenitor cells in hypoxia-induced pulmonary hypertensive rats. Eur. J. Pharmacol..

[43] Ghouleh I.A., Sahoo S., Meijles D.N., Amaral J.H., de Jesus D.S., Sembrat J., Rojas M., Goncharov D.A., Goncharova E.A., Pagano P.J. (2017). Endothelial Nox1 oxidase assembly in human pulmonary arterial hypertension; driver of Gremlin1-mediated proliferation. Clin. Sci..

[44] Ban Y., Liu Y., Li Y., Zhang Y., Xiao L., Gu Y., Chen S., Zhao B., Chen C., Wang N. (2019). S-nitrosation impairs KLF4 activity and instigates endothelial dysfunction in pulmonary arterial hypertension. Redox Biol..

[45] Ochoa C.D., Wu R.F., Terada L.S. (2018). ROS signaling and ER stress in cardiovascular disease. Mol. Asp. Med..

[46] Kikuchi N., Satoh K., Kurosawa R., Yaoita N., Elias-Al-Mamun M., Siddique M.A.H., Omura J., Satoh T., Nogi M., Sunamura S. (2018). Selenoprotein P Promotes the Development of Pulmonary Arterial Hypertension. Circulation.

[47] Pena E., Brito J., El Alam S., Siques P. (2020). Oxidative Stress, Kinase Activity and Inflammatory Implications in Right Ventricular Hypertrophy and Heart Failure under Hypobaric Hypoxia. Int. J. Mol. Sci..

[48] Shults N., Melnyk O., Suzuki D., Suzuki Y. (2018). Redox Biology of Right-Sided Heart Failure. Antioxidants.

[49] Stam K., Cai Z., van der Velde N., van Duin R., Lam E., van der Velden J., Hirsch A., Duncker D.J., Merkus D. (2019). Cardiac remodelling in a swine model of chronic thromboembolic pulmonary hypertension: Comparison of right vs. left ventricle. J. Physiol..

[50] Ren X., Johns R.A., Gao W.D. (2019). Right heart in pulmonary hypertension: From adaptation to failure. Pulm. Circ..

[51] Boehm M., Novoyatleva T., Kojonazarov B., Veit F., Weissmann N., Ghofrani H.A., Seeger W., Schermuly R.T. (2019). Nitric Oxide Synthase 2 Induction Promotes Right Ventricular Fibrosis. Am. J. Respir. Cell Mol. Biol..

[52] Wang D., Li H., Weir E.K., Xu Y., Xu D., Chen Y. (2019). Dimethylarginine dimethylaminohydrolase 1 deficiency aggravates monocrotaline-induced pulmonary oxidative stress, pulmonary arterial hypertension and right heart failure in rats. Int. J. Cardiol..

[53] Rudyk O., Rowan A., Prysyazhna O., Krasemann S., Hartmann K., Zhang M., Shah A.M., Ruppert C., Weiss A., Schermuly R.T. (2019). Oxidation of PKGIα mediates an endogenous adaptation to pulmonary hypertension. Proc. Natl. Acad. Sci. USA.

[54] Ayinapudi K., Singh T., Motwani A., Le Jemtel T.H., Oparil S. (2018). Obesity and Pulmonary Hypertension. Curr. Hypertens. Rep..

[55] Hansen T., Galougahi K.-K., Celermajer D., Rasko N., Tang O., Bubb K.J., Figtree G. (2016). Oxidative and nitrosative signalling in pulmonary arterial hypertension—Implications for development of novel therapies. Pharmacol. Ther..

[56] Larissi K., Politou M., Margeli A., Poziopoulos C., Flevari P., Terpos E., Papassotiriou I., Voskaridou E. (2019). The Growth Differentiation Factor-15 (GDF-15) levels are increased in patients with compound heterozygous sickle cell and beta-thalassemia (HbS/βthal), correlate with markers of hemolysis, iron burden, coagulation, endothelial dysfunction and pulmonary hypertension. Blood Cells Mol. Dis..

[57] Xu T., Shao L., Wang A., Liang R., Lin Y., Wang G., Zhao Y., Hu J., Liu S. (2020). CD248 as a novel therapeutic target in pulmonary arterial hypertension. Clin. Transl. Med..

[58] Weise-Cross L., Resta T.C., Jernigan N.L. (2019). Redox Regulation of Ion Channels and Receptors in Pulmonary Hypertension. Antioxid. Redox Signal..

[59] Mathew R. (2020). Signaling Pathways Involved in the Development of Bronchopulmonary Dysplasia and Pulmonary Hypertension. Children.

[60] Fallah F. (2015). Recent Strategies in Treatment of Pulmonary Arterial Hypertension, A Review. Glob. J. Health Sci..

[61] Feng W., Hu Y., An N., Feng Z., Liu J., Mou J., Hu T., Guan H., Zhang D., Mao Y. (2020). Alginate Oligosaccharide Alleviates Monocrotaline-Induced Pulmonary Hypertension via Anti-Oxidant and Anti-Inflammation Pathways in Rats. Int. Heart J..

[62] Kang Y., Zhang G., Huang E.C., Huang J., Cai J., Cai L., Wang S., Keller B.B. (2020). Sulforaphane prevents right ventricular injury and reduces pulmonary vascular remodeling in pulmonary arterial hypertension. Am. J. Physiol. Heart Circ. Physiol..

[63] Mansouri Z., Dianat M., Radan M., Badavi M. (2020). Ellagic Acid Ameliorates Lung Inflammation and Heart Oxidative Stress in Elastase-Induced Emphysema Model in Rat. Inflammation.

[64] Gonzalez-Candia A., Veliz M., Carrasco-Pozo C., Castillo R.L., Cárdenas J.C., Ebensperger G., Reyes R.V., Llanos A.J., Herrera E.A. (2019). Antenatal melatonin modulates an enhanced antioxidant/pro-oxidant ratio in pulmonary hypertensive newborn sheep. Redox Biol..

[65] Astorga C.R., González-Candia A., Candia A.A., Figueroa E.G., Cañas D., Ebensperger G., Reyes R.V., Llanos A.J., Herrera E.A. (2018). Melatonin Decreases Pulmonary Vascular Remodeling and Oxygen Sensitivity in Pulmonary Hypertensive Newborn Lambs. Front. Physiol..

[66] Zhang M., Chang Z., Zhao F., Zhang P., Hao Y.-J., Yan L., Liu N., Wang J.-L., Bo L., Ma P. (2019). Protective Effects of 18β-Glycyrrhetinic Acid on Monocrotaline-Induced Pulmonary Arterial Hypertension in Rats. Front. Pharmacol..

[67] Budas G.R., Boehm M., Kojonazarov B., Viswanathan G., Tian X., Veeroju S., Novoyatleva T., Grimminger F., Hinojosa-Kirschenbaum F., Ghofrani H.A. (2018). ASK1 Inhibition Halts Disease Progression in Preclinical Models of Pulmonary Arterial Hypertension. Am. J. Respir. Crit. Care Med..

[68] Boucherat O., Provencher S., Bonnet S. (2018). Therapeutic Value of ASK1 Inhibition in Pulmonary Arterial Hypertension. Am. J. Respir. Crit. Care Med..

[69] Lampron M.C., Vitry G., Nadeau V., Grobs Y., Paradis R., Samson N., Tremblay E., Boucherat O., Meloche J., Bonnet S. (2020). PIM1 (Moloney Murine Leukemia Provirus Integration Site) Inhibition Decreases the Nonhomologous End-Joining DNA Damage Repair Signaling Pathway in Pulmonary Hypertension. Arterioscler. Thromb. Vasc. Biol..

[70] Kurosawa R., Satoh K., Kikuchi N., Kikuchi H., Saigusa D., Al-Mamun M.E., Siddique M.A.H., Omura J., Satoh T., Sunamura S. (2019). Identification of Celastramycin as a Novel Therapeutic Agent for Pulmonary Arterial Hypertension. Circ. Res..

[71] Becker C.U., Sartório C.L., Campos-Carraro C., Siqueira R., Colombo R., Zimmer A., Belló-Klein A. (2020). Exercise training decreases oxidative stress in skeletal muscle of rats with pulmonary arterial hypertension. Arch. Physiol. Biochem..

[72] Fan Y.-F., Zhang R., Jiang X., Wen L., Wu D.-C., Liu D., Yuan P., Wang Y.-L., Jing Z.-C. (2013). The phosphodiesterase-5 inhibitor vardenafil reduces oxidative stress while reversing pulmonary arterial hypertension. Cardiovasc. Res..

[73] Steven S., Oelze M., Brandt M., Ullmann E., Kröller-Schön S., Heeren T., Tran L.P., Daub S., Dib M., Stalleicken D. (2017). Pentaerythritol Tetranitrate In Vivo Treatment Improves Oxidative Stress and Vascular Dysfunction by Suppression of Endothelin-1 Signaling in Monocrotaline-Induced Pulmonary Hypertension. Oxid. Med. Cell. Longev..

[74] Dianat M., Radan M., Mard S.A., Sohrabi F., Saryazdi S.S.N. (2020). Contribution of reactive oxygen species via the OXR1 signaling pathway in the pathogenesis of monocrotaline-induced pulmonary arterial hypertension: The protective role of Crocin. Life Sci..

[75] Xu D., Li Y., Zhang B., Wang Y., Liu Y., Luo Y., Niu W., Dong M., Liu M., Dong H. (2016). Resveratrol alleviate hypoxic pulmonary hypertension via anti-inflammation and anti-oxidant pathways in rats. Int. J. Med. Sci..

[76] Sunamura S., Satoh K., Kurosawa R., Ohtsuki T., Kikuchi N., Elias-Al-Mamun M., Shimizu T., Ikeda S., Suzuki K., Satoh T. (2018). Different roles of myocardial ROCK1 and ROCK2 in cardiac dysfunction and postcapillary pulmonary hypertension in mice. Proc. Natl. Acad. Sci. USA.

[77] Cheng Y., Gong Y., Qian S., Mou Y., Li H., Chen X., Kong H., Xie W., Wang H., Zhang Y. (2018). Identification of a Novel Hybridization from Isosorbide 5-Mononitrate and Bardoxolone Methyl with Dual Activities of Pulmonary Vasodilation and Vascular Remodeling Inhibition on Pulmonary Arterial Hypertension Rats. J. Med. Chem..

[78] Li T., Li S., Feng Y., Zeng X., Dong S., Li J., Zha L., Luo H., Zhao L., Liu B. (2020). Combination of Dichloroacetate and Atorvastatin Regulates Excessive Proliferation and Oxidative Stress in Pulmonary Arterial Hypertension Development via p38 Signaling. Oxid. Med. Cell. Longev..

[79] Shi R., Wei Z., Zhu D., Fu N., Wang C., Yin S., Liang Y., Xing J., Wang X., Wang Y. (2018). Baicalein attenuates monocrotaline-induced pulmonary arterial hypertension by inhibiting vascular remodeling in rats. Pulm. Pharmacol. Ther..

[80] Liu Z., Duan Y.L., Ge S.L., Zhang C.X., Gong W.H., Xu J.J. (2019). Effect of estrogen on right ventricular remodeling of monocrotaline-induced pulmonary arterial hypertension in rats and its mechanism. Eur. Rev. Med. Pharmacol. Sci..

[81] Campos-Carraro C., Turck P., de Lima-Seolin B.G., Tavares A.M.V., Dos Santos Lacerda D., Corssac G.B., Teixeira R.B., Hickmann A., Llesuy S., da Rosa Araujo A.S. (2018). Copaiba Oil Attenuates Right Ventricular Remodeling by Decreasing Myocardial Apoptotic Signaling in Monocrotaline-Induced Rats. J. Cardiovasc. Pharmacol..

[82] Grzegorzewska A.P., Seta F., Han R., Czajka C.A., Makino K., Stawski L., Isenberg J.S., Browning J.L., Trojanowska M. (2017). Dimethyl Fumarate ameliorates pulmonary arterial hypertension and lung fibrosis by targeting multiple pathways. Sci. Rep..

[83] de Lima-Seolin B.G., Hennemann M.M., Fernandes R.O., Colombo R., Bonetto J.H.P., Teixeira R.B., Khaper N., Godoy A.E.G., Litvin I.E., Sander da Rosa Araujo A. (2018). Bucindolol attenuates the vascular remodeling of pulmonary arteries by modulating the expression of the endothelin-1 A receptor in rats with pulmonary arterial hypertension. Biomed. Pharmacother..

[84] DeMarco V.G., Habibi J., Whaley-Connell A.T., Schneider R.I., Sowers J.R., Andresen B.T., Gutweiler A.A., Ma L., Johnson M.S., Ferrario C.M. (2009). Rosuvastatin ameliorates the development of pulmonary arterial hypertension in the transgenic (mRen2)27 rat. Am. J. Physiol. Heart Circ. Physiol..

[85] Zhang Q., Fan K., Wang P., Yu J., Liu R., Qi H., Sun H., Cao Y. (2016). Carvacrol induces the apoptosis of pulmonary artery smooth muscle cells under hypoxia. Eur. J. Pharmacol..

[86] Türck P., Lacerda D.S., Carraro C.C., de Lima-Seolin B.G., Teixeira R.B., Poletto Bonetto J.H., Colombo R., Schenkel P.C., Belló-Klein A., da Rosa Araujo A.S. (2018). Trapidil improves hemodynamic, echocardiographic and redox state parameters of right ventricle in monocrotaline-induced pulmonary arterial hypertension model. Biomed. Pharmacother..

[87] Wang X., Yang Y., Yang D., Tong G., Lv S., Lin X., Chen C., Dong W. (2016). Tetrandrine prevents monocrotaline-induced pulmonary arterial hypertension in rats through regulation of the protein expression of inducible nitric oxide synthase and cyclic guanosine monophosphate-dependent protein kinase type 1. J. Vasc. Surg..

[88] Liu B., Luo X.-J., Yang Z.-B., Zhang J.-J., Li T.-B., Zhang X.-J., Ma Q.-L., Zhang G.-G., Hu C.-P., Peng J. (2014). Inhibition of NOX/VPO1 pathway and inflammatory reaction by trimethoxystilbene in prevention of cardiovascular remodeling in hypoxia-induced pulmonary hypertensive rats. J. Cardiovasc. Pharmacol..

[89] Kishimoto Y., Kato T., Ito M., Azuma Y., Fukasawa Y., Ohno K., Kojima S. (2015). Hydrogen ameliorates pulmonary hypertension in rats by anti-inflammatory and antioxidant effects. J. Thorac. Cardiovasc. Surg..

[90] Türck P., Fraga S., Salvador I., Campos-Carraro C., Lacerda D., Bahr A., Ortiz V., Hickmann A., Koetz M., Belló-Klein A. (2020). Blueberry extract decreases oxidative stress and improves functional parameters in lungs from rats with pulmonary arterial hypertension. Nutrition.

[91] Meghwani H., Prabhakar P., Mohammed S., Dua P., Seth S., Hote M., Banerjee S., Arava S., Ray R., Maulik S. (2018). Beneficial Effect of Ocimum sanctum (Linn) against Monocrotaline-Induced Pulmonary Hypertension in Rats. Medicines.

[92] Black S.M., Nozik-Grayck E. (2019). Compartmentalization of Redox-Regulated Signaling in the Pulmonary Circulation. Antioxid. Redox Signal..

[93] Spiekerkoetter E., Kawut S.M., de Jesus Perez V.A. (2019). New and Emerging Therapies for Pulmonary Arterial Hypertension. Annu. Rev. Med..

[94] Walsh T., Baird G., Atalay M., Agarwal S., Arcuri D., Klinger J., Mullin C., Morreo H., Normandin B., Shiva S. (2021). Experimental design of the Effects of Dehydroepiandrosterone in Pulmonary Hypertension (EDIPHY) trial. Pulm. Circ..

[95] Wu Y., Mi Y., Zhang F., Cheng Y., Wu X. (2021). Suppression of bromodomain-containing protein 4 protects trophoblast cells from oxidative stress injury by enhancing Nrf2 activation. Hum. Exp. Toxicol..

[96] Valenca S., Dong B., Gordon E., Sun R., Waters C. (2022). ASK1 Regulates Bleomycin-induced Pulmonary Fibrosis. Am. J. Respir. Cell Mol. Biol..

